# BMC Medical Informatics and Decision Making

**DOI:** 10.1186/1472-6947-14-7

**Published:** 2014-01-29

**Authors:** Irene Pala

**Affiliations:** 1 BioMed Central, 236 Gray’s Inn, Road, London WC1X 8HB, UK

## Abstract

**Contributing reviewers:**

The editors of BMC Medical Informatics and Decision Making would like to thank all our reviewers who have contributed their time to the journal in Volume 13 (2013).

## 

Samantha Adams

Netherlands

Anil Agarwal

USA

Nazim Agoulmine

France

Ankit Agrawal

USA

Raheelah Ahmad

UK

John Ainsworth

UK

Paul Albert

USA

Urs-Vito Albrecht

Germany

José F Aldana

Spain

K Rivet Amico

USA

Milena Anatchkova

USA

Jessica Ancker

USA

Robert Anderson

USA

Marine Andersson

Sweden

Thomas Aparicio

France

Carmel Apicella

Australia

Shams Arifeen

Bangladesh

Joanna Armstrong Schellenberg

UK

Knut Magne Augestad

Norway

Daniel Austin

USA

Alexander Avian

Austria

Haleh Ayatollahi

UK

Steven Babin

USA

Joonbum Bae

Korea South

James Baggs

USA

Gianluca Baio

UK

Mark Ballermann

Canada

Samir Bandyopadhyay

India

Adrian Barbu

USA

Jennifer Barton

USA

Melissa Baysari

Australia

Jason Beckstead

USA

Cosmin Bejan

USA

Hilary Bekker

UK

Ewert Bengtsson

Sweden

Carol Bennett

Canada

Adrian Benton

USA

Ugur Bilge

Turkey

Roger Bjugn

Norway

David Blazes

USA

Megan Bohensky

Australia

Carol Bond

UK

Damian Borbolla

USA

Alessio Bottrighi

Italy

Matt-Mouley Bouamrane

UK

Christopher Bowd

USA

James Boyd

Australia

Teresa Brady

USA

Johann Brink

Canada

Anna L Buczak

USA

Howard Burkom

USA

Hongmin Cai

China

Peter Callery

UK

G Iain Carpenter

UK

Joao Paulo Carvalho

Portugal

Maaret Castren

Sweden

Andrea Cereatti

Italy

Wendy Chapman

USA

Bruno Charpiat

France

Kun-Huang Chen

Taiwan

Shih-Chih Chen

Taiwan

Yu-Chun Chen

Taiwan

Qiang Cheng

USA

Aswani Kumar Cherukuri

India

Zaida Chinchilla-Rodríguez

Spain

Hung-Wen Chiu

Taiwan

Ya-Wen Chiu

Taiwan

Peter Christen

Australia

Jen-Hsiang Chuang

Taiwan

John Chuo

USA

James Cimino

USA

Luca Citi

UK

Karen Clark

USA

Isabelle Colombet

France

Craig Conover

USA

Matthew Cooperberg

USA

Giuliana Cortese

Italy

Francisco Couto

Portugal

Nigel William Crawford

Australia

Kathrin Cresswell

UK

Jesse Crosson

USA

Ricardo Cruz-Correia

Portugal

Elizabeth Cummings

Australia

Mollie Cummins

USA

Solveig Cunningham

USA

Stefan Darmoni

France

Sudeshna Das

USA

Andrew Davidson

Australia

Sandra De Groote

USA

Thyra de Jongh

Netherlands

Fabrizio De Ponti

Italy

Lukasz Debowski

Poland

Ewa Deelman

USA

Mathaeus Dejori

USA

Lucia Del Vecchio

Italy

Nico Dellaert

Netherlands

Bart Demaerschalk

USA

Lesley Diack

UK

Sharmila Dissanaike

USA

James Dolan

USA

Jenny Doust

Australia

Ulrich Ebner-Priemer

Germany

Ruth Endacott

UK

Saeid Eslami

Netherlands

Martine Extermann

USA

Jim Fackler

USA

Oliver Faust

Singapore

Arild Faxvaag

Norway

Joaquim Cezar Felipe

Brazil

Susan Fenton

USA

Jesualdo Tomas Fernandez-Breis

Spain

Oscar Ferrandez

USA

Jan Florin

Sweden

Juliane Fluck

Germany

Uno Fors

Sweden

Hamish Fraser

USA

Clark Freifeld

USA

Noreen Frisch

Canada

Virgile Fritsch

France

Dominick Frosch

USA

Marie-Pierre Gagnon

Canada

Bill Galanter

USA

Omar Galarraga

USA

Barbara Gandek

USA

Sebastian Garde

Germany

Bruno Gaume

France

Rolf Gedeborg

Sweden

Andrew Georgiou

Australia

Philippe Giabbanelli

Canada

Mireille Goetghebeur

Canada

William Goossen

Netherlands

Natalia Grabar

France

Kathleen Gray

Australia

Michelle Greiver

Canada

Yulong Gu

New Zealand

Kenneth Guappone

USA

Stephanie Guerlain

USA

Saleema Gulzar

Pakistan

William Gunning

USA

Steven Guthridge

Australia

Patricia Halfon

Switzerland

Brett Hauber

USA

Sarah Hawley

USA

Gillian Hayes

USA

Inga Hege

Germany

Ulrike Held

Switzerland

Annemaeri Hellebek

Denmark

David Hepner

USA

Vitaly Herasevich

USA

Marlien Herselman

South Africa

Thomas Higgins

USA

Lisa Hines

USA

Oliver Hirsch

Germany

Harry Hochheiser

USA

Richard Holden

USA

Bernhard Holzner

Austria

Carol Homko

USA

Michael Horberg

USA

Simon Horsburgh

New Zealand

Jan Horsky

USA

George Hsieh

USA

Jiin-Chyr Hsu

Taiwan

Jina Huh

USA

Linda Huibers

Netherlands

Ollivier Hyrien

USA

Sylvia Hysong

USA

Turgay Ibrikci

Turkey

Jan Illing

UK

Ivan Ip

USA

Eloy Irigoyen

Spain

Conrade Carl Jaffe

USA

Monique Jaspers

Netherlands

Tewa Jean Jules

Cameroon

Alexander Jetter

Switzerland

Xiaoqian Jiang

USA

Masahito Jimbo

USA

Antonio Jimeno Yepes

USA

Kari Johansen

Sweden

kerina jones

UK

Loic Josseran

France

Stan Kachnowski

USA

Ananth Kalyanaraman

USA

Devan Kansagara

USA

Murat Kantarcioglu

USA

Dimitris Karlis

Greece

Monika Kastner

Canada

Maria Katapodi

USA

Richard Katz

USA

Kensaku Kawamoto

USA

Nurten Kaya

Turkey

Justin Keen

UK

Harry Kennedy

Ireland

Kate Khair

UK

Serkan Kiranyaz

Finland

Jeffrey Klann

USA

Michael Klompas

USA

Sabine Koch

Sweden

Walter Koller

Austria

Patrick kolsteren

Belgium

Gary Kreps

USA

Sunil Kripalani

USA

Jeffrey Kullgren

USA

Tyge-F Kummer

Australia

Ichiro Kunitsugu

Japan

Alex Kuo

Canada

Laurie Lachance

USA

Faustina Lalatta

Italy

Benjamin Lamb

UK

Jean-Baptiste Lamy

France

Marek Laskowski

Canada

Francis Lau

Canada

Caroline Laurence

Australia

Michaël Laurent

Belgium

Ting-Ting Lee

Taiwan

France Légaré

Canada

Luigi Lepanto

Canada

William D Leslie

Canada

Guangxi Li

USA

Rong-Ho Lin

Taiwan

Andreas Linninger

USA

Chiung-ju Liu

USA

Gunnar Ljunggren

Sweden

Paolo Locatelli

Italy

Craig Locatis

USA

Ellen Lopez

USA

Miguel López-Coronado

Spain

Grigorios Loukides

UK

Kimberly Lovett

USA

Xudong Lu

China

Sheng Luo

USA

Courtney Lyles

USA

Jason Lyman

USA

Velio Macellari

Italy

Vijay Mago

USA

Jose Alberto Maldonado

Spain

Stephen Maloney

Australia

Kenneth Mandl

USA

Ronny Mans

Netherlands

Marianthi Markatou

USA

Joel Martin

Canada

Linda Gore Martin

USA

Roshan Martis

Singapore

Andrew Masica

USA

Carl May

UK

Erin McClure

USA

Allison McCoy

USA

Paul McCullagh

UK

Carolyn McGregor

Canada

Jorit Meesters

Netherlands

David Menotti

Brazil

Sally Merry

New Zealand

Stephane Meystre

USA

Ellen M Mikkelsen

Denmark

Jose Joaquin Mira

Spain

Hiroki Mishina

Japan

Marc Mitchell

USA

Anne Moen

Norway

Pavitra Mohan

India

Rafael Morales-Bueno

Spain

Allisyn Moran

USA

Lulu Muhe

Switzerland

Hillary Mull

USA

Maria Pilar Muñoz

Spain

Wilbroad Mutale

Zambia

Radhakrishnan Nagarajan

USA

Kim Nazi

USA

Huong Nguyen

USA

Michael B Nichol

USA

Wendy Nilsen

USA

Frederick North

USA

Natasha Noy

USA

Olumuyiwa Odusanya

Nigeria

Jose Luis Oliveira

Portugal

Margaret Olsen

USA

Wail Omar

Canada

Brenda O'Neill

UK

Cristina Oroviogoicoechea

Spain

Rema Padman

USA

Timothy Page

USA

Massimiliano Panella

Italy

Supasit Pannarunothai

Thailand

George Panoutsos

UK

Bev Paterson

Australia

Christopher Pearce

Australia

Alan Pearson

Australia

Sallie Pearson

Australia

Niels Peek

Netherlands

Christine Phillips

Australia

Michael Pine

USA

Ednaldo Pizzolato

Brazil

Pierre Pluye

Canada

Klaus Pommerening

Germany

Giampiero Porzio

Italy

Mikaela Poulymenopoulou

Greece

Justin Presseau

UK

Jialan Que

USA

Brian Quilliam

USA

Peter Raich

USA

Padmanabhan Ramnarayan

UK

Mark Rapoport

Canada

Theo Raynor

UK

Gérard Reach

France

Nicola Reavley

Australia

Eva Rebelo Gomes

Portugal

Andrew Reisner

USA

Emilie Renahy

Canada

Patricia Riley

USA

Heleen Riper

Netherlands

Pedro Pereira Rodrigues

Portugal

Michelle Rogers

USA

Michael Rosato

UK

David Rovner

USA

Abraham Rudnick

Canada

Alissa Russ

USA

Stefan Russmann

Switzerland

Carolyn Rutter

USA

Paul Rutter

UK

Keun Ho Ryu

Korea South

David Sanchez

Spain

Javier Sanz-Valero

Spain

Sharon Saydah

USA

Anne-Marie Scheepers-Hoeks

Netherlands

Christos Schizas

Cyprus

Peter J Schulz

Switzerland

Gerold Schwantzer

Austria

Martin Sedlmayr

Germany

Hanna Seidling

Germany

Hossein Seif Zadeh

Australia

Carlo Senore

Italy

Shaouli Shahid

Australia

Nicola Shaw

Canada

Jay Shen

USA

Christopher Sherlaw-Johnson

UK

Kerry Sherman

Australia

Qian Shi

USA

Sarah Shih

USA

Matthew Shotwell

USA

Mark Siedner

USA

Magenta Simmons

Australia

Davide Sisti

Italy

Dean Sittig

USA

Marina Sokolova

Russian Federation

Anthony Solomonides

USA

Vishal Sondhi

India

Lixin Song

USA

Mohamed Sorror

USA

Lina F Soualmia

France

Emmanouil Spanakis

Greece

Dawn Stacey

Canada

Sven Stieglitz

Germany

Gregor Stiglic

Slovenia

Michael Stoto

USA

Sandeep Subramanian

Canada

Brian Suffoletto

USA

Hilary Surratt

USA

Michelle Sweidan

Australia

Jeffrey Switzer

USA

Maria Taboada

Spain

Tassos TAGARIS

Greece

Suzanne Tamang

USA

Xin Tang

USA

Leonor Teixeira

Portugal

Tommi Tervonen

Netherlands

Cindy Thomas

USA

Carl Thompson

UK

William Thompson

USA

Sally Thorne

Canada

Lili Tian

USA

Luca Toldo

Germany

Andrew Tomarken

USA

Vicenc Torra

Spain

Lyndal Trevena

Australia

Gianluca Trifiro

Italy

Traian Truta

USA

Allan Tucker

UK

Aris Tzavaras

Greece

Shahadat Uddin

Australia

John Valenza

USA

Bert-Jan Van Beijnum

Netherlands

Ben Van Calster

Belgium

Geert JMG van der Heijden

Netherlands

Lian van der Krieke

Netherlands

Maja van der Velden

Norway

Matthijs van Leeuwen

Belgium

Alies van Lier

Netherlands

Maaike van Mourik

Netherlands

Erik van Mulligen

Netherlands

Tjeerd van Staa

UK

Mirjam van Veen

Netherlands

robert Vander Stichele

Belgium

Robert Veltri

USA

Suzanne Verstappen

UK

Marcia Vervloet

Netherlands

Vassilios Verykios

Greece

Joshua Vest

USA

Vivian Vimarlund

Sweden

Sholom Wacholder

USA

Kavishwar Wagholikar

USA

Nuwan Waidyanatha

China

Jeen-Shing Wang

Taiwan

Shuang Wang

USA

Jim Warren

New Zealand

Griffin Weber

USA

Wei Wei

USA

Charlene Weir

USA

Kuang-Yi Wen

USA

Chunhua Weng

USA

Duminda Wijesekera

USA

W John Wilbur

USA

Margaret Williamson

Australia

Deborah Winn

USA

Nancy Winslade

Canada

Holly Witteman

Canada

Fredric Wolf

USA

Dennis Wollersheim

Australia

Yonghui Wu

USA

Yuan Wu

USA

Til Wykes

UK

Po-Yin Yen

USA

Illhoi Yoo

USA

Bob Young

UK

Jiang Yu

USA

Ping Yu

Australia

Vesna Zeljkovic

USA

Daniel Zeng

USA

Ning Zhang

USA

Yungang Zhang

UK

Kai Zheng

USA

## Pre-publication history

The pre-publication history for this paper can be accessed here:

http://www.biomedcentral.com/1472-6947/14/7/prepub

